# Prevalence of gastrointestinal helminth infections in ovine population of Kashmir Valley

**DOI:** 10.14202/vetworld.2015.1199-1204

**Published:** 2015-10-17

**Authors:** S. R. Tramboo, R. A. Shahardar, I. M. Allaie, Z. A. Wani, M. S. Bushra

**Affiliations:** Division of Veterinary Parasitology, Faculty of Veterinary Science and Animal Husbandry, Sher-E-Kashmir University of Agricultural Sciences and Technology of Kashmir, Shuhama Campus, Alusteng, Srinagar - 190 006, Jammu and Kashmir, India

**Keywords:** coproculture, gastrointestinal, Kashmir, nemathelminths, ovine, prevalence, platyhelminths

## Abstract

**Aim:**

Gastrointestinal (GI) helminth parasitism is one of the major constraints for profitable sheep production. Due to variations in the prevalence of GI helminths from region to region, it becomes important to map out accurately the parasitic fauna of a particular region for development of suitable control measures.

**Materials and Methods:**

An extensive study of GI helminths was carried out in Budgam district of Kashmir Valley over a period of 1 year. A total of 1200 fresh ovine faecal samples from both sexes of young ones and adults were collected in sterilized plastic bags and examined by standard sedimentation and floatation techniques. Positive faecal samples (15-20%) in each season were examined by Stoll’s dilution method to determine the parasitic load. A total of 120 faecal samples (30 samples in each season) positive for strongyle eggs were subjected to coproculture using Petridish method and the third stage larvae were harvested to find out prevalence of different genera of strongyle worms.

**Results:**

The overall prevalence of GI helminths was found to be 77% with platyhelminths and nemathelminthes in 26.58 and 60.92% animals, respectively. The overall prevalence of mixed GI helminths was found to be 8.67%. Eggs of various helminths encountered in the present study were those of *Fasciola* spp. (3.58%), *Dicrocoelium* spp. (11.58%), paramphistomes (4.83%), *Moniezia* spp. (7.92%), strongyle worms including *Nematodirus* spp. (57.75%), *Strongyloides* spp. (1.67%), and *Trichuris* spp. (1.5%). On coprocultural examination *Haemonchus* spp. (55%) was found to be most predominant strongyle worm followed by *Trichostrongylus* spp. (17.5%), *Ostertagia* spp. (11.67%), *Oesophagostomum* spp. (9.17%), and *Chabertia* spp. (6.67%). On seasonal basis, highest prevalence of GI helminths was recorded in summer (83.00%) followed by spring (78.67%), winter (76.33%), and autumn (70.00%), the difference being statistically non-significant (p>0.05). The prevalence of platyhelminths (*Fasciola* spp., *Dicrocoelium* spp. and *Moniezia* spp.) was found to be non-significantly higher in winter, but paramphistomes showed the highest prevalence in the summer season. Nemathelminth infection was found highest in summer season and lowest during the winter season. Eggs per gram (EPG) ranged from 0 to 1800, and an average EPG count was found to be 454.35±27.85. EPG was found to be highest in summer (684.00±69.83) and lowest in winter (202.38±18.82). The overall prevalence of GI helminths was found more in adult sheep (83.00%) compared to young ones (53.11%), the difference being statistically significant (p<0.05). Similarly, the prevalence of helminths was found to be higher in females (78.32%) as compared to males (72.97%), the variation being statistically non-significant (p>0.05).

**Conclusion:**

Seasonal variation plays an important role in the prevalence of GI helminths in addition to age and sex of the animal.

## Introduction

Sheep sufficing a multipurpose need of wool, milk, skin, meat, and manure is the main source of income to the marginal farmers of the country [1]. Sheep are highly susceptible to gastrointestinal (GI) parasitosis, thereby inflicting losses through morbidity, mortality, reduced feed conversion ratio, poor wool or meat quality, and by way of costs incurred on treatment and control [2]. In India, helminth diseases alone are responsible for 5% mortality and more than 10% morbidity in sheep [3]. The overall development of the rural hilly areas could not be achieved by neglecting the development of the agricultural commodities such as sheep and goats [4].

The prevalence of these GI helminths varies from region to region depending upon the local climatic conditions of the region and managemental practices adopted. As such the parasitic fauna of each region should be mapped out accurately for the development of various control measures. In Kashmir Valley, the incidence of parasitic infection in sheep has been reported by various workers [4-11] but these studies are restricted to either organized farms or to sheep flocks in and around Srinagar city, and no such study have been carried out in locally reared sheep of Budgam district in particular.

In our study, GI parasitism in ovine population was thoroughly screened over a period of 1 year in all the tehsils of Budgam district of Kashmir Valley.

## Materials and Methods

### Ethical approval

The present study was based on the laboratory examination of faeces of sheep and no experiment was conducted on the animals. So, permission is not necessary for such type of study. However, samples were collected as per standard sample collection procedure.

### Study area

Budgam is bounded by Baramulla and Srinagar in the north, Pulwama in the south and Poonch border in the southwest. It is situated at an average altitude of 5281 ft above mean sea level and at 75° East longitude and 34° North latitude. The topography of the district is mixed with both mountainous and plain areas. Administratively, this district is divided into six tehsils namely Chari - Sharief, Beerwah, Budgam, Chadoora, Khag, and Khan sahib and covers an area of 1,371 Km^2^. The climate is of the temperate type with the upper reaches receiving heavy snowfall in winter. The maximum temperature varies from 12.83°C to 34.17°C, and minimum temperature varies from −4.47°C to 12.13°C. Total rainfall and relative humidity varies between 35.87 and 90.33 mm and 66.74 and 80.18%, respectively.

### Sample collection and processing

A total of 1200 samples were collected from all the six tehsils of district Budgam for a period of 1 year. The samples were collected directly from rectum and brought to the laboratory in mini zip locked polythene bags for examination. During collection, name of the owner, place of collection, age, and sex of the animals were recorded. On the basis of age, animals were classified as adults (age > 1 year) and young (age < 1 year). These samples were collected during all the four seasons *viz*.; summer (June-August), autumn (September-November), winter (December-February), and spring (March-May) with 300 samples in each season. Samples were preserved at refrigeration temperature (4°C) till examination and examined within 2-3 days after collection. The samples were examined qualitatively using sedimentation and floatation techniques for evaluating the incidence of infection [12]. Randomly selected 15-20% faecal samples were examined in each season by Stoll’s dilution method to determine the parasitic load, i.e. eggs per gram (EPG) of faeces [12]. A total of 120 faecal samples (30 samples in each season) positive for strongyle eggs were subjected to coproculture using petridish method [13] for harvesting third stage larvae. The harvested larvae were examined using key morphological differences described by Van-Wyk *et al*. [14] to work out the prevalence of different genera of strongyle worms.

### Statistical analysis

The results were subjected to standard statistical analysis as per Snedecor and Cochran [15].

## Results and Discussion

In this study, the overall prevalence of GI helminths in sheep of Budgam district was found to be 77.00% with mixed infection in 8.67% animals (Table-1). Average EPG was found to be 454.35±27.85 (EPG range: 0-1800) (Table-2). On qualitative examination, low level of strongyle worm infection was observed, which on quantitative examination (Stolls dilution method) revealed zero (0) EPG. Almost similar incidence has been reported by Pandit *et al*. [6] (65.4% overall prevalence) and Bhat *et al*. [11] (62.9%overall prevalence) in sheep of Kashmir Valley and Yadav *et al*. [16] (83.24% overall prevalence) and Khajuria *et al*. [17] (68.54% overall prevalence) in sheep of Jammu, respectively. In this study, the overall prevalence of platyhelminths was 26.58% with trematodes and cestodes having a prevalence of 19.67% and 7.92%, respectively. Prevalence of *Dicrocoelium* spp. (11.58%) was highest followed by paramphistomes (4.83%), while *Fasciola* spp. (3.58%) had the lowest prevalence. *Moniezia* spp. was the only cestode parasite found in the present study with a percentage prevalence of 7.92 (Table-1). Our observations are almost similar to Shahnawaz *et al*. [9] who reported an overall prevalence of platyhelminths to be 28.50% with trematodes and cestodes in 18.66% and 11.83% sheep respectively in Ganderbal district of Kashmir Valley. The overall prevalence of nemathelminths in this study was 60.92% with a maximum prevalence of Strongyle worms (57.75%) followed by *Strongyloides* spp. (1.67%) and *Trichuris* spp. (1.50%) (Table-1). The results of the present investigation are in close approximation to the findings of Tariq *et al*. [8] who reported 61.6% overall prevalence of nemathelminth parasites in sheep of Kashmir Valley. Predominance of strongyle group of worms over other nematodes noted in this study has also been reported by Pandit *et al*. [6,7] and Wani *et al*. [10] in sheep of Kashmir Valley as well as Khajuria and Kapoor [18] and Yadav *et al*. [16] in sheep of Jammu region. Kumar *et al*. [19] reported strongyle worm infection as a major cause of GI tract disorders of sheep followed by *Strongyloides* spp. in Sheep Breeding Farm, Lala Lajpat Rai University of Veterinary and Animal Sciences, Hisar, Haryana.

**Table-1:** Overall and Seasonal prevalence of gastrointestinal helminth parasites of sheep in district Budge of Kashmir Valley.

Season	Number of samples	Mixed infection	F	D	P	Total trematodes	M	Total cestodes	Total platyhelminths	S	St	T	Total nematodes	Total GI helminths
Summer	300	23 (7.67^a^)	3 (1.00^a^)	26 (8.67^a^)	25 (8.33^a^)	53 (17.67^a^)	22 (7.33^a^)	22 (7.33^a^)	73 (24.33^ab^)	191 (63.67^a^)	7 (2.33^a^)	5 (1.67^a^)	203 (67.67^a^)	249 (83.00^a^)
Autumn	300	13 (4.33^a^)	9 (3.00^a^)	16 (5.33^a^)	17 (5.67^a^)	42 (14.00^a^)	15 (5.00^a^)	15 (5.00^a^)	57 (19.00^a^)	166 (55.33^a^)	3 (1.00^a^)	4 (1.33^a^)	173 (57.67^a^)	210 (70.00^a^)
Winter	300	36 (12.00^a^)	18 (6.00^a^)	56 (18.67^a^)	6 (2.00^a^)	79 (26.33^a^)	35 (11.67^a^)	35 (11.67^a^)	107 (35.67^b^)	155 (51.67^a^)	5 (1.67^a^)	5 (1.67^a^)	165 (55.00^a^)	229 (76.33^a^)
Spring	300	32 (10.67^a^)	13 (4.33^a^)	41 (13.67^a^)	10 (3.33^a^)	62 (20.67^a^)	23 (7.67^a^)	23 (7.67^a^)	82 (27.33^ab^)	181 (60.33^a^)	5 (1.67^a^)	4 (1.33^a^)	190 (63.33^a^)	236 (78.67^a^)
Total	1200	104 (8.67)	43 (3.58)	139 (11.58)	58 (4.83)	236 (19.67)	95 (7.92)	95 (7.92)	319 (26.58)	693 (57.75)	20 (1.67)	18 (1.50)	731 (60.92)	924 (77.00)

Figures within parenthesis indicate percentage; Values with the same superscript in a column under a subgroup do not vary significantly (p>0.05), Where, F=*Fasciola* spp., D=*Dicrocoelium* spp., P=Paramphistomes, M=*Moniezia* spp., S=Strongyle worms, St=*Strongyloides* spp., T=*Trichuris* spp., GI=Gastrointestinal,

a,b : Means having different superscription in row differ significantly

**Table-2:** Age-wise and seasonal comparison of a parasitic load of GI helminth parasites of sheep in Budgam district of Kashmir.

Season	Host	Number of samples screened	EPG range	Mean EPG
Summer	Adult	31	0-1700	754.84±70.46
	Young	19	0-1800	568.42±142.26
	Total	50	0-1800	684.00^a^±69.83
Autumn	Adult	28	0-500	321.43±25.90
	Young	16	0-500	262.50±49.05
	Total	44	0-500	300.00^b^±24.33
Winter	Adult	27	0-300	211.11±22.85
	Young	15	0-300	186.67±33.62
	Total	42	0-300	202.38^b^±18.82
Spring	Adult	32	0-900	587.50±61.69
	Young	16	0-800	556.25±81.12
	Total	48	0-800	577.08^a^±48.76
Overall	Adult	118	0-1700	482.20^a^±32.56
	Young	66	0-1800	404.55^a^±51.16
	Total	184	0-1800	454.35±27.85

EPG: Eggs per gram,

a,b : Means having different superscription in row differ significantly

**Figure-1:**
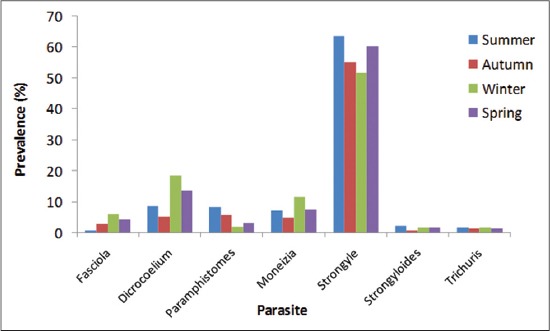
Seasonal prevalence of gastrointestinal helminths of sheep in Budgam, Kashmir.

Non-significantly (p>0.05) prevalence of GI helminths was found highest in summer season (83.00%) followed by spring (78.67%), winter (76.33%), and lowest in autumn (70.00%). The higher platyhelminth infection rate was found in winter (35.67%) followed by spring (27.33%), summer (24.33%), and autumn (19%). However, the difference was significant (p<0.05) between autumn and winter seasons. In this study, the highest infection rate of nemathelminths was observed in summer (67.67%) followed by spring (63.33%), autumn (57.67%), and winter (55%) (Table-1). Average EPG was found highest in summer season, 684±69.83 (EPG range: 0-1800) followed by spring 577.08±48.76 (EPG range: 0-800); autumn 300.00±24.33 (EPG range: 0-500), and winter 202.38±18.82 (EPG range: 0-300), the variation being statistically significant (p<0.05) (Table-2). This observation goes in agreement with Swarnkar *et al*. [20] who observed peak parasitism in the summer season. Yadav *et al*. [16] and Sharma *et al*. [21] also observed the highest EPG in rainy season with the lowest EPG in winter season in sheep of Jammu and Palam Valley of north western Himalayan region, respectively. The highest rate of infection in summer is due to very favorable environmental conditions (temperature - maximum 34.17°C, minimum 12.13°C; 35.87 mm rainfall, and 66.74% relative humidity) for hatching of eggs and development of free living larval stages. In spring (temperature - maximum 25.0°C, minimum 2.0°C; 75.30 mm rainfall, and 67.14% relative humidity), and autumn (temperature - maximum 27.33°C, minimum 1.57°C; 49.37 mm rainfall and 71.92% relative humidity), due to moderate temperature hatching and development of free living larval stages gets comparatively delayed and in winter (temperature - maximum 12.83°C, minimum −4.47°C; 90.33 mm rainfall, and 80.18% relative humidity) due to extremely low environmental temperature, hypobiosis occurs and therefore there is reduction in faecal egg counts. Trematodes showed a similar trend with the highest prevalence of 26.33% in winter followed by 20.67%, 17.67%, and 14.00% in spring, summer, and autumn, respectively, seasonal variation being non-significant (p>0.05) except for paramphistomes which showed a different seasonal activity with the highest prevalence of 8.33% in summer followed by 5.67%, 3.33%, and 2.00% in autumn, spring, and winter, respectively (Table-1 and Figure-1). The highest prevalence of *Fasciola* spp. in winter is due to grazing of sheep in harvested paddy fields during autumn after return from alpine pastures where they pick up the infection and worms reach to sexual maturity during winter and thereby, start passing eggs in faeces. The higher prevalence rate of paramphistomes during summer season is logistic since during the drier months snail population becomes concentrated around areas of natural water which also have the most palatable grazing, thus there is concentration of snails, metacercariae, and animals over a small area leading to heavy infection [12]. Godara *et al*. [22] also recorded a higher percentage of sheep positive for paramphistomes in autumn season as compared to winter. Non-significantly (p>0.05), *Moniezia* spp. was found to be highest in winter (11.67%) followed by spring (7.67%), summer (7.33%), and autumn (5%) (Table-1 and Figure-1).

**Figure-2:**
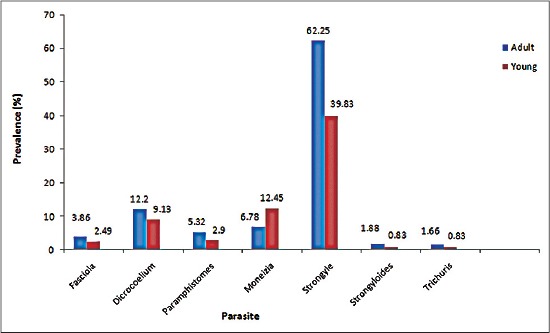
Age-wise prevalence of gastrointestinal helminthes of sheep in Budgam, Kashmir.

Significantly, (p<0.05) higher prevalence rate of GI helminths (83.00%) was observed in adult sheep compared to lambs and hoggets (53.11%). The higher prevalence rate of trematodes was observed in adult sheep compared to lambs and hoggets, the difference being statistically non-significant (p>0.05). However, *Moniezia* spp. infection was more prevalent in the younger group (12.45%) as against 6.78% in adult age group, the difference being statistically non-significant (p>0.05). Nemathelminths were found higher in adult sheep (65.80%) in comparison to young ones (41.49%), the variation being statistically significant (p<0.05) (Figure-2). Non-significantly (p>0.05), EPG was found higher in adult sheep (482.20±32.56) as compared to young sheep (404.55±51.16) (Table-2). Our observations of higher EPG in adult sheep as compared to young sheep are in agreement with those of Swarankar *et al*. [20] and Yadav *et al*. [16] who reported higher prevalence of helminths in adult sheep compared to young ones in Rajasthan and Jammu, respectively. Shahnawaz *et al*. [9] also reported higher trematode infections in adults as compared to young sheep but higher *Moniezia* infection in lambs as compared to adult sheep. The highest prevalence of helminth parasites in adult animals compared to young ones is due to their long exposure to infective stages in grazing fields and also because the sheep reared by farmers are not properly dosed or underdosed which results in build-up of infection. Low prevalence of *Moniezia* spp. in adults compared to young ones in the present study may be attributed to previous infection and age of host which affords some protection against reinfection, thereby causing severe monieziosis in young animals [12]. Wani *et al*. [10] has also reported a higher prevalence of nemathelminths in adult sheep compared to young ones in Ganderbal district of Kashmir Valley.

**Figure-3:**
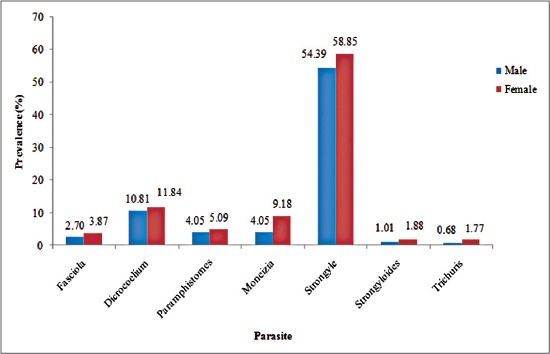
Sex-wise prevalence of gastrointestinal helminths of sheep in Budgam, Kashmir.

The prevalence of helminth parasites recorded in this study *viz. Fasciola* spp. (2.7% in males and 3.87% in females), *Dicrocoelium* spp. (10.81% in males and 11.84% in females), paramphistomes (4.05% in males and 5.09% in females), *Moniezia* spp. (4.05% in males and 9.18% in females), strongyle worms (54.39% in males and 58.85% in females), *Strongyloides* spp. (1.01% in males and 1.88% in females), and *Trichuris* spp. (0.68% in males and 1.77% in females) were higher in females compared to males, the variation being statistically non-significant (p>0.05) (Figure-3). The higher infection rate of parasites in female sheep are in line with Mbap and Chiroma [23] who found female sheep more susceptible to infection than males. The higher infection rate of platyhelminths and nemathelminths in females has also been reported by Shahnawaz *et al*. [9] and Wani *et al*. [10], respectively in Ganderbal district of Kashmir Valley. Significantly, (p<0.05) an average EPG (490.65±32.64) was found to be higher in females than males (342.22±49.90). The reason can be attributed to post parturition and lactation stress leading to high shedding of eggs of GI nematodes, thereby increasing pasture infectivity. In lactating animals, there is an increase in susceptibility of ewes to newly acquired the infection during the periparturient period [24]. Highest prevalence (18.67%) of *Dicrocoelium* spp. in winter has also been reported by Shahnawaz *et al*. [9] and can be attributed to the fact that animals pick up the infection during the summer and autumn seasons from paddy fields and apple orchids and the parasites reach to sexual maturity in winter season.

On coprocultural examination of strongyle worms, *Haemonchus* spp. (55.00%) was found to be most predominant strongyle worm followed by *Trichostrongylus* spp. (17.50%)*, Ostertagia* spp. (11.67%), *Oesophagostomum* spp. (9.17%), and *Chabertia* spp. (6.67%). The predominance of *Haemonchus* spp. over other strongyle worms has also been reported by Tariq *et al*. [8] and Wani *et al*. [10] in Kashmir Valley. Domke *et al*. [25] and Kuchai *et al*. [26] also recorded *Haemonchus* to be one of the most prevalent strongyle worms in sheep from Norway and Ladakh respectively. Singh *et al*. [27] reported *Haemonchus* as the main GI parasite in sheep and goats in and around Mathura, India. The reason for this predominance can be due to the fact that *Haemonchus* females are basically prolific egg layers (laying about 10,000 eggs a day).

## Conclusion

The present communication documents prevalence of GI helminths in sheep of Budgam district of Kashmir Valley. Seasonal variation plays an important role in the prevalence of GI helminths in addition to age and sex of the animal. The overall high prevalence of gastrointestinal helminth infection (77%) in Budgam district of Kashmir Valley can be attributed to poor managemental techniques that are being practiced by the local farmers. Besides poor managemental practices, lack of pastures in the valley makes the animals to graze the same land time and again, thereby resulting in building of infections in these animals. Also, animals were not previously dewormed, and lack of knowledge of proper dosing methods by the sheep owners keep infection rate high in these animals. Based on the research findings of this study, it is concluded that the broad spectrum anthelmintics which are effective against both platyhelminths and nemathelminths may be used in prophylactic treatment of animals twice (early spring and late autumn) or thrice in a year (early spring, late summer and late autumn) to prevent production losses in the Budgam district of Kashmir Valley.

## Authors’ Contributions

SRT processed out the samples and prepared the manuscript under the guidance of RAS and IMA. RAS designed the study. IMA provided technical support and gave final shape to the manuscript. ZAW and BMS provided manual help. All authors read and approved the final manuscript.
